# Guanidinylation of Chitooligosaccharides Involving Internal Cyclization of the Guanidino Group on the Reducing End and Effect of Guanidinylation on Protein Binding Ability

**DOI:** 10.3390/biom9070259

**Published:** 2019-07-05

**Authors:** Hironori Izawa, Mizuki Kinai, Shinsuke Ifuku, Minoru Morimoto, Hiroyuki Saimoto

**Affiliations:** 1 Graduate School of Engineering, Tottori University, 4-101 Koyama-Minami, Tottori 680-8550, Japan; 2 Centre for Research on Green Sustainable Chemistry, Tottori University, Tottori 680-8550, Japan; 3 Division of Instrumental Analysis, Research Center for Bioscience and Technology, Tottori University, Tottori 680-8550, Japan

**Keywords:** chitooligosaccharide, guanidinylation, 1-amidinopyrazole hydrochloride, reducing end, bovine serum albumin, cyclic guanidine

## Abstract

In order to synthesize a promising material for developing a novel peptide/protein delivery system, guanidinylation of chitooligosaccharides with 1-amidinopyrazole hydrochloride was investigated herein. The production of guanidinylated chitooligosaccharides was demonstrated by infrared spectroscopy (IR), nuclear magnetic resonance (NMR), and elemental analyses. Interestingly, we found that the reducing end in the guanidinylated chitooligosaccharides was converted to a cyclic guanidine structure (2-[(aminoiminomethyl)amino]-2-deoxy-d-glucose structure). This reaction was carefully proven by the guanidinylation of d-glucosamine. Although this is not the first report on the synthesis of the 2-[(aminoiminomethyl)amino]-2-deoxy-d-glucose, it has provided a rational synthetic route using the high reactivity of the reducing end. Furthermore, we found that the interaction between chitooligosaccharides and bovine serum albumin is weak when in a neutral pH environment; however, it is significantly improved by guanidinylation. The guanidinylated chitooligosaccharides are useful not only for the development of a novel drug delivery system but also as a chitinase/chitosanase inhibitor and an antibacterial agent.

## 1. Introduction

Chitosan (CS) is an amino polysaccharide, composed of β-d-glucosamine (>60%) and β-d-*N*-acetylglucosamine linking through a β-1,4-linkage and obtained by the deacetylation of chitin [1]. Chitosans with degree of polymerization (DP) < 20 and that have an average molecular weight of up to 3900 Da are called chitooligosaccharides (COSs) [2]. Although common chitosans are insoluble in a neutral water, COSs are highly soluble. In addition, COSs are reported to possess remarkable biological properties [3,4], undoubtedly making them attractive materials for developing novel pharmaceuticals.

Improvement of the low biomembrane permeability of peptide/protein drugs is a key aspect of pharmaceutical sciences [5]. Cell-penetrating peptide (CPP) has attracted great attention as a promising material for delivering such drugs into cells [6]. However, their cytotoxicity and the innate immune response problem has limited their applications in clinics [7]. In addition, in the case of oral administration, CPPs consisting of L-amino acids are often unable to realize their potential because they are digested in the digestive tract [8].

We reported facile preparation of guanidinylated chitosan with 1-amidinopyrazole hydrochloride (AP) [9] because the guanidinylation of chitosan would enhance the biomembrane permeability of peptide/protein drugs, similar to the effect of arginine-rich CPPs [5,6,10,11], and the lower acidity of the protonated guanidino group (pKa = 12.5) would enhance the electrostatic interaction with protein/peptide drugs in a neutral pH environment [12]. Besides that, we confirmed enhanced interaction between the guanidinylated chitosan and protein at acidic and neutral pHs. Application of this method to COSs would provide guanidinylated COS (GCOS), a promising material for developing a novel peptide/protein delivery system. In addition, GCOS would have lower toxicity and an indigestibility suitable for oral administration because COSs have good biocompatibility and indigestibility.

On the other hand, protection-free synthesis for making carbohydrate-related compounds has attracted great attention as it allows us to be released from the laborious protection–deprotection routes. The reducing end showing high reactivity is an attractive target to achieve a protection-free synthesis of valuable building blocks. The production of glycosyl amines [13], glycosyl azides [14], and glycosyl donors [15,16,17], and the immobilization of carbohydrate building block via reductive amination [18], by virtue of the high reactivity of the reducing end, are widely employed in carbohydrate chemistry. The development of protection-free synthesis for making building blocks that still require the protection–deprotection process is effective for developing medicines, agrochemicals, and functional materials. 2-[(Aminoiminomethyl)amino]-2-deoxy-d-glucose, having a cyclic guanidine structure on the reducing end, was expected as a catalytic cofactor analogue for the *glmS* ribozyme [19]. It is expected to have inhibitory activity toward chitinase/chitosanase because of the structural similarity to chitin/chitosan. However, there are no synthetic routes that do not involve protection–deprotection procedures.

**Scheme 1:**
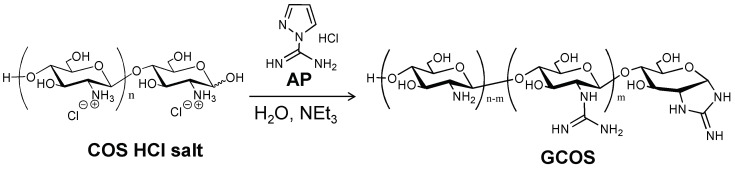
Guanidinylation of chitooligosaccharides (COS) with 1-amidinopyrazole hydrochloride (AP).

Herein, we investigated the guanidinylation of a COS with AP (Scheme 1). Interestingly, we found that GCOS has a cyclic guanidine structure on the reducing end. In addition, the effects of the guanidinylation on protein binding ability were investigated with fluorescence analysis using bovine serum albumin (BSA) as a model protein drug.

## 2. Materials and Methods 

### 2.1. Materials

COS hydrochloride with an undeacetylated 4.7% fraction (elemental analysis) (monomer 0.3%, dimer 12.8%, trimer 25.3%, tetramer 27.1%, pentamer 24.2%, hexamer 5.9%, heptamer 2.1%, and octamer 1.0% in weight) was supplied from Koyo Chemical (Tottori, Japan). 1-Amidinopyrazole hydrochloride (AP), d-glucosamine hydrochloride, acetic anhydride, and pyridine were purchased from Tokyo Chemical Industry (Tokyo, Japan). Bovine serum albumin (BSA) (>98%) was purchased from the Sigma-Aldrich (St. Louis, MO, USA). Other reagents were used as received.

### 2.2. Instruments

Nuclear magnetic resonance (NMR) spectra were recorded on a JNM-ECP500 (JEOL, Tokyo, Japan) or an Avance II600 (Bruker, Billerica, MA, USA). Infrared (IR) spectra of the samples were measured using a Spectrum 65 (Perkin-Elmer Japan, Tokyo, Japan) equipped with an attenuated total reflection (ATR) attachment. Elemental analysis data were measured on a Perkin Elmer 2400 II CHNS/O (Perkin Elmer, Franklin Lakes, NJ, USA). Mass spectra were measured using an Exactive^TM^ plus Orbitrap MASS spectrometer (Thermo Fischer Scientific, Waltham, MA, USA). Fluorescence spectra were recorded on an RF-6000 (Shimadzu, Kyoto, Japan).

### 2.3. Preparation of Guanidinylated Chitooligosaccharides

Chitooligosaccharides (COS) hydrochloride (1.00 g), AP (2.70 g, 18.4 mmol), and trimethylamine (3.34 g, 33.0 mmol) were dissolved in water, and the mixture was stirred for seven days at room temperature. The reaction mixture was poured into a large volume of isopropanol. The precipitated product was collected by filtration, followed by washing with methanol. The product was dried under reduced pressure. The yield (product (g)/theoretical maximum yield (g) × 100) was 26.8% (0.275 g).

### 2.4. Preparation of Guanidinylated Chitooligosaccharides Sulfate Salt

Chitooligosaccharides (COS) hydrochloride (1.00 g), AP (2.70 g, 18.4 mmol), and trimethylamine (3.34 g, 33.0 mmol) were dissolved in water, and the mixture was stirred for seven days at room temperature. The reaction mixture was poured into a large volume of isopropanol, and the precipitated product was collected by filtration. The precipitate was dissolved in a 1.0 M H_2_SO_4_ aqueous solution (2 mL). The solution was poured into a large volume of methanol, and the precipitated COS sulfate salt was collected by filtration and then washed with methanol. This operation for producing sulfate salt was repeated. The product was dried under reduced pressure. The yield was 50.2% (0.575 g). Elemental analysis: C/H/N/S = 30.70:5.99:11.24:6.11.

### 2.5. Guanidinylation and Subsequent Acetylation of d-Glucosamine

Glucosamine hydrochloride (1.00 g, 4.64 mmol), AP (0.67 g, 4.64 mmol), and trimethylamine (1.28 g, 9.20 mmol) were added to water, and the mixture was stirred for one day at room temperature. The reaction mixture was lyophilized and the lyophilized material was added to pyridine (10 mL). Acetic anhydride (9.40 g, 92.1 mmol) was added to the solution, and the mixture was stirred for one day. The reaction solution was poured into 1.0 M HCl aqueous solution. The acetylated product was extracted with chloroform. The chloroform layer was washed with a 1.0 M HCl aqueous solution three times and then dried with Na_2_SO_4_. After the chloroform layer was evaporated, the residue was purified by silica gel chromatography (hexane/ethyl acetate = 2:1).

### 2.6. Fluorescence Analysis on Interaction between Guanidinylated Chitooligosaccharides and Bovine Serum Albumin

The quenching constant *K* (M^−1^), between COS or GCOS and BSA, was calculated from a Stern–Volmer plot [20]:

(1) $$\frac{F_{0}}{F}=K\left[Total\;unit\right]+1,$$

where *F* and *F*_0_ is the maximum fluorescence intensity of the BSA solution in the presence and absence of COS or GCOS, respectively.

## 3. Results and Discussion

### 3.1. Preparation of Guanidinylated Chitooligosaccharides

**Figure 1:**
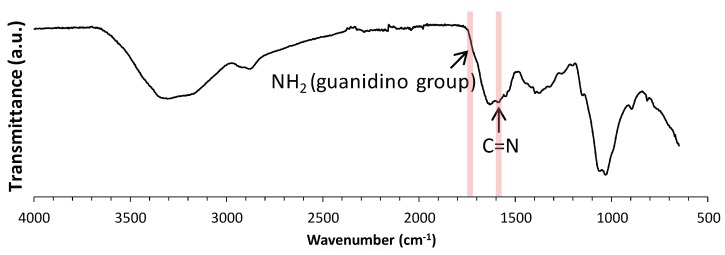
Infrared (IR) spectrum of guanidinylated chitooligosaccharide (GCOS).

**Figure 2:**
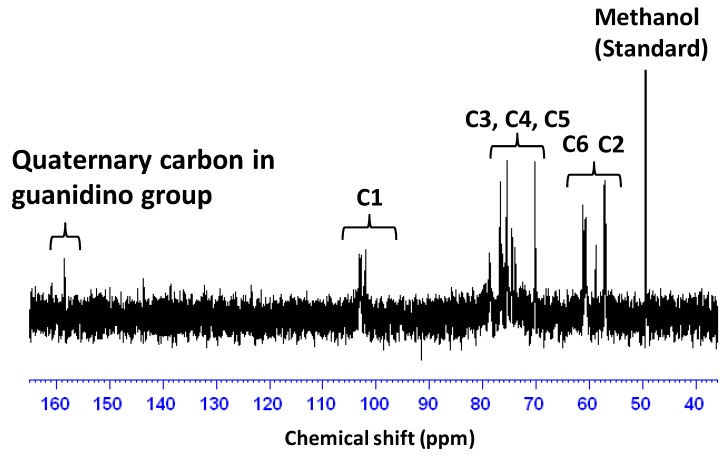
^13^C NMR spectrum of GCOS in D_2_O. This spectrum was measured in the presence of methanol as an internal standard. The signal due to methanol was adjusted to 49.5 ppm [23].

Guanidinylation was performed with 3.8 equivalents (equiv) of AP toward an amino group in COS at room temperature for seven days [9]. The product was isolated as a methanol-insoluble fraction at a 26.8% yield. Figure 1 shows the IR spectrum of the product. In the spectrum, the characteristic absorption peaks, attributed to the C=N stretching vibration and the NH bending vibration of guanidino groups [9], were observed at around 1725 cm^−1^ and 1590 cm^−1^, respectively. Figure 2 shows the ^13^C NMR spectrum of the product. In the spectrum, the signals, due to the quaternary carbon in the guanidino groups, are shown at around 159.3 ppm [9,21,22]. These results clearly indicate the production of GCOS. The degree of guanidinylation (guanidinylated units (mol)/total units (mol) × 100) was 66.3%, as estimated by elemental analysis (C/N = 4.22).

**Figure 3:**
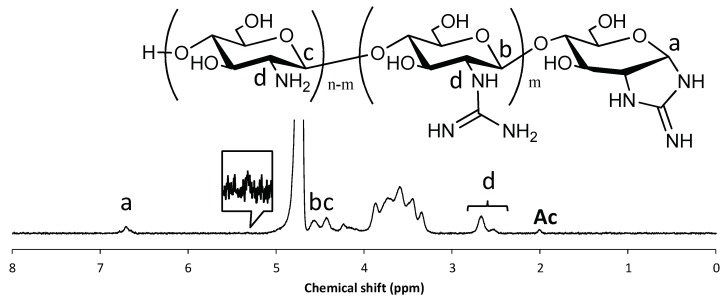
^1^H NMR spectrum of GCOS in D_2_O. The signal attributed to residual acetyl groups was adjusted to 2.0 ppm.

**Scheme 2:**
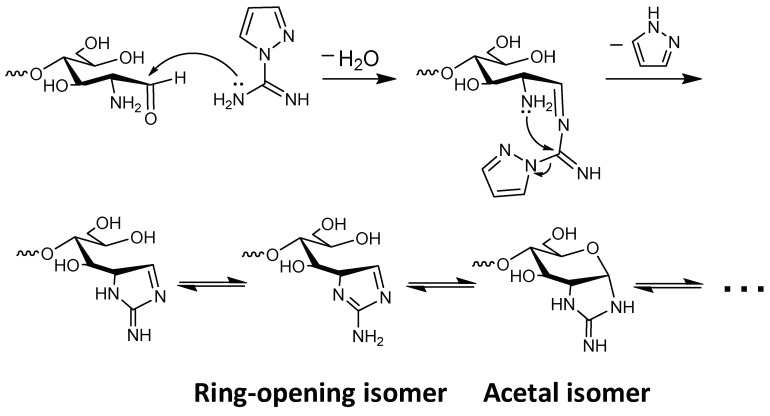
Plausible mechanism of cyclization on the reducing end.

In the ^1^H NMR spectrum, an unexpected signal was observed at 6.74 ppm (Figure 3). In addition, the signal attributed to the anomeric proton on the reducing end (α-isomer), observed at 5.37 ppm in the spectrum of COS hydrochloride (Figure S9), was hardly observed. These results suggest that the newly appeared signal was attributable to the anomeric proton on the reducing end. We conjectured that efficient internal cyclization of the guanidino group via Schiff base production [18] (Scheme 2) had occurred, leading to formation of a cyclic guanidino moiety. Indeed, the chemical shift of the signal attributed to the anomeric proton on the reducing end (Figure 3) was consistent with that of its reported analog (2-[(aminoiminomethyl)amino]-2-deoxy-d-glucose-6-phosphate prepared via multistep organic synthesis) [19]. The signal attributed to the proton of the 2-position on the reducing end was observed at around 4.6 ppm on the H–H COSY spectra (Figure S2). This chemical shift also agreed with that of its reported analog [19]. In addition, in the electrospray ionization-mass spectrometry (ESI-MS) spectrum, the detected molecular ion peaks showed good agreement with dehydrated GCOS (Figure S1). These results strongly suggested the production of a cyclic guanidine structure. Note that the low resolution of the ^1^H NMR spectrum (Figure 3) is probably due to strong aggregation of GCOS by the guanidinylation.

**Scheme 3:**
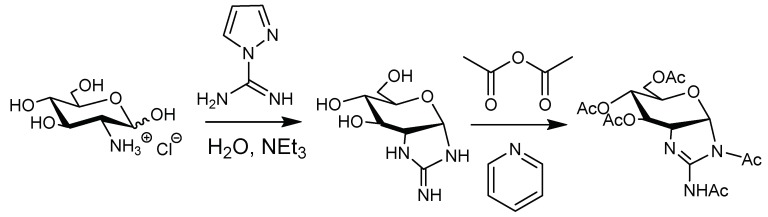
Guanidinylation and subsequent acetylation of GlcN.

In order to prove that a cyclic guanidine structure was produced, the guanidinylation of d-glucosamine (GlcN) was investigated (Scheme 3). Guanidinylation was carried out with 1.0 equiv of AP at room temperature for 24 h (Figure S4). The ESI–MS analysis of the lyophilized reaction mixture showed a molecular ion peak (*m/z* = 204.098) corresponding to protonated 2-[(aminoiminomethyl)amino]-2-deoxy-d-glucose (calc. C_7_H_14_N_3_O_4_^+^ = 204.10) (Figure S5). The lyophilized mixture was acetylated and purified by silica gel chromatography. The ^1^H NMR and ESI–MS analysis showed the production of 2-[(aminoiminomethyl)amino]-2-deoxy-d-glucose and/or its isomers after guanidinylation (Figures S6–S8). Note that although the cyclic guanidine structure on the reducing end is described herein as an acetal isomer, we suppose that it presents as a ring-opening isomer because the signal attributed to the anomeric proton was detected in the olefin region, as shown in Figure 3, and had not disappeared under acidic or basic conditions (1.0 M DCl or NaOD solution, respectively) (Figure S3).

The low yield of GCOS, isolated as the methanol-insoluble fraction, was attributable to the high solubility of GCOS in methanol, i.e., a large quantity of GCOS was removed, along with impurities, during the purification process. In order to overcome the low yield, GCOS was isolated as sulfate salt by precipitation with methanol, where the yield was up to 50.2%. The structure of GCOS sulfate salt was confirmed by the IR and ^1^H NMR analyses (Figures S10 and S11, respectively). The degree of guanidinylation of GCOS sulfate salt was 54.0%, which was lower than that of the GCOS as described before. This is probably due to the difference in the solubility of GCOS depending on the degree of guanidinylation, i.e., highly guanidinylated GCOS showed lower solubility in methanol. Indeed, desalted COS, used here, is soluble in methanol. Namely, GCOS, having a lower degree of guanidinylation, could be collected by being sulfate salt, leading to the higher yield.

### 3.2. Fluorescence Analysis on Interaction between Guanidinylated Chitooligosaccharides and Bovine Serum Albumin

**Figure 4:**
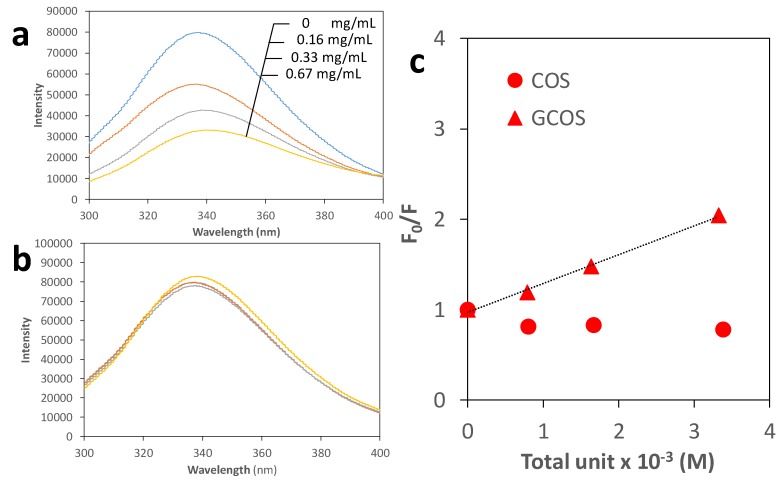
Fluorescence spectra (excitation wavelength: 275 nm) of 0.42 mg/mL BSA in the presence of 0, 0.16, 0.33, 0.67 mg/mL GCOS (0, 0.79, 1.64, 3.32 × 10^−3^ M, respectively) or COS (0, 0.81, 1.67, 3.39 × 10^−3^ M, respectively) in a 0.1 M Tris–HCl buffer (pH 7.4) (**a** or **b**, respectively) and their Stern–Volmer plots (**c**).

Comparison of the protein binding ability between GCOS and COS was investigated with fluorescence analysis using BSA, where the interaction between GCOS or COS and BSA can be estimated from the quenching behavior of fluorescence owing to Trp, Tyr, and Phe residues [24,25,26,27]. Figure 4a and b shows the fluorescence spectra of 0.42 mg/mL BSA in the presence of 0–0.67 mg/mL of either GCOS or COS in a neutral pH, respectively. In the case of GCOS, fluorescence emission peaks gradually quenched by increasing the quantity of GCOS [28]. By contrast, fluorescence emission peaks were not changed when COS increased, suggesting the interaction between COS and BSA was very weak. This result clearly indicates the interaction between GCOS and BSA was remarkably enhanced by the guanidinylation. The quenching constant between GCOS and BSA estimated from the slope of the Stern–Volmer plots (Figure 4c) was 318 M^–1^. This is comparable to that of chitosan in acidic conditions [9].

## 4. Conclusions

In conclusion, GCOS was prepared by the guanidinylation of COS with AP. Interestingly, the cyclic guanidino moiety was produced on the reducing end during the reaction. Although this is not the first report on the synthesis of the 2-[(aminoiminomethyl)amino]-2-deoxy-d-glucose structure, it has provided a rational synthetic route using the high reactivity of the reducing end. In addition, GCOS could be isolated in moderate yield as the GCOS sulfate salt. Besides that, we investigated the effect of guanidino groups on the protein binding ability in a neutral pH with fluorescence analysis using BSA. Fluorescence quenching was not observed by increasing the quantity of COS, while the fluorescence was gradually quenched with GCOS increases, clearly indicating remarkable enhancement of the protein binding ability by guanidinylation. Evaluation of the cytotoxicity and biomembrane permeability of GCOS is now in progress. The GCOS is useful not only for the development of a novel drug delivery system but also as a chitinase/chitosanase inhibitor [29] and an antibacterial agent [21,30].

## Supplementary Materials

The following are available online at https://www.mdpi.com/2218-273X/9/7/259/s1, Figure S1: ESI–MS spectra of GCOS, Figure S2: H–H COSY NMR spectra of GCOS in D_2_O, Figure S3: ^1^H NMR spectra of GCOS in 1.0 M NaOD and DCl–D_2_O solutions after 2 days, Figure S4: ^1^H NMR spectrum of the lyophilized mixture in D_2_O , Figure S5: ESI–MS spectrum obtained on the positive mode of the lyophilized mixture, Figure S6: ESI–MS spectrum obtained on the positive mode of the acetylated product, Figure S7: ^1^H NMR spectra of the acetylated product in CDCl_3_, Figure S8: ^1^H NMR spectrum of COS hydrochloride, Figure S10: IR NMR spectrum of the GCOS sulfate salt, Figure S11. ^1^H NMR spectrum of the GCOS sulfate salt.

## References

[1] Younes I., Rinaudo M. (2015). Chitin and Chitosan Preparation from Marine Sources. Structure, Properties and Applications. Mar. Drugs.

[2] Mourya V.K., Inamdar N.N., Choudhari Y.M. (2011). Chitooligosaccharides: Synthesis, Characterization and Applications. Polym. Sci. Ser. A.

[3] Muanprasat C., Chatsudthipong V. (2017). Chitosan oligosaccharide: Biological activities and potential therapeutic applications. Pharm. Ther..

[4] Zou P., Yang X., Wang J., Li Y.F., Yu H.L., Zhang Y.X., Liu G.Y. (2016). Advances in characterisation and biological activities of chitosan and chitosan oligosaccharides. Food Chem..

[5] Kristensen M., Nielsen H.M. (2016). Cell-Penetrating Peptides as Carriers for Oral Delivery of Biopharmaceuticals. Basic Clin. Pharmacol. Toxicol..

[6] Khafagy E.S., Morishita M. (2012). Oral biodrug delivery using cell-penetrating peptide. Adv. Drug Deliv. Rev..

[7] Uusna J., Langel K., Langel U. (2015). Toxicity, Immunogenicity, Uptake, and Kinetics Methods for CPPs. Methods Mol. Biol..

[8] Kamei N., Shigei C., Hasegawa R., Takeda-Morishita M. (2018). Exploration of the Key Factors for Optimizing the in Vivo Oral Delivery of Insulin by Using a Noncovalent Strategy with Cell-Penetrating Peptides. Biol. Pharm. Bull..

[9] Izawa H., Kinai M., Ifuku S., Morimoto M., Saimoto H. (2019). Guanidinylated chitosan inspired by arginine-rich cell-penetrating peptides. Int. J. Biol. Macromol..

[10] Murayama T., Masuda T., Afonin S., Kawano K., Takatani-Nakase T., Ida H., Takahashi Y., Fukuma T., Ulrich A.S., Futaki S. (2017). Loosening of Lipid Packing Promotes Oligoarginine Entry into Cells. Angew. Chem. Int. Ed..

[11] Takeuchi T., Futaki S. (2016). Current Understanding of Direct Translocation of Arginine-Rich Cell-Penetrating Peptides and Its Internalization Mechanisms. Chem. Pharm. Bull..

[12] Mogaki R., Hashim P.K., Okuro K., Aida T. (2017). Guanidinium-based ’molecular glues’ for modulation of biomolecular functions. Chem. Soc. Rev..

[13] Norris P. (2008). Pyranose N-glycosyl amines: Emerging targets with diverse biological potential. Curr. Top. Med. Chem..

[14] Tanaka T., Nagai H., Noguchi M., Kobayashi A., Shoda S. (2009). One-step conversion of unprotected sugars to beta-glycosyl azides using 2-chloroimidazolinium salt in aqueous solution. Chem. Commun..

[15] Alexander S.R., Lim D., Amso Z., Brimble M.A., Fairbanks A.J. (2017). Protecting group free synthesis of glycosyl thiols from reducing sugars in water; application to the production of N-glycan glycoconjugates. Org. Biomol. Chem..

[16] Noguchi M., Kobayashi A., Shoda S. (2015). The One-step Preparation of Sugar Oxazoline Enables the Synthesis of Glycoprotein Having a Definite Structure. Trends Glycosci. Glyc..

[17] Noguchi M., Tanaka T., Gyakushi H., Kobayashi A., Shoda S. (2009). Efficient Synthesis of Sugar Oxazolines from Unprotected N-Acetyl-2-amino Sugars by Using Chloroformamidinium Reagent in Water. J. Org. Chem..

[18] Ruhaak L.R., Steenvoorden E., Koeleman C.A.M., Deelder A.M., Wuhrer M. (2010). 2-Picoline-borane: A non-toxic reducing agent for oligosaccharide labeling by reductive amination. Proteomics.

[19] Posakony J.J., Ferre-D’Amare A.R. (2013). Glucosamine and Glucosamine-6-phosphate Derivatives: Catalytic Cofactor Analogues for the glmS Ribozyme. J. Org. Chem..

[20] Keizer J. (1983). Non-Linear Fluorescence Quenching and the Origin of Positive Curvature in Stern-Volmer Plots. J. Am. Chem. Soc..

[21] Hu Y., Du Y.M., Yang J.H., Kennedy J.F., Wang X.H., Wang L.S. (2007). Synthesis, characterization and antibacterial activity of guanidinylated chitosan. Carbohydr. Polym..

[22] Zhai X.Y., Sun P., Luo Y.F., Ma C.N., Xu J., Liu W.G. (2011). Guanidinylation: A Simple Way to Fabricate Cell Penetrating Peptide Analogue-Modified Chitosan Vector for Enhanced Gene Delivery. J. Appl. Polym. Sci..

[23] Gottlieb H.E., Kotlyar V., Nudelman A. (1997). NMR chemical shifts of common laboratory solvents as trace impurities. J. Org. Chem..

[24] Bekale L., Agudelo D., Tajmir-Riahi H.A. (2015). Effect of polymer molecular weight on chitosan-protein interaction. Colloid Surf. B.

[25] Li G., Huang J.Y., Chen T., Wang X.Y., Zhang H.J., Chen Q. (2017). Insight into the interaction between chitosan and bovine serum albumin. Carbohydr. Polym..

[26] Rabbani G., Baig M.H., Jan A.T., Lee E.J., Khan M.V., Zaman M., Farouk A.E., Khan R.H., Choi I. (2017). Binding of erucic acid with human serum albumin using a spectroscopic and molecular docking study. Int. J. Biol. Macromol..

[27] Rabbani G., Lee E.J., Ahmad K., Baig M.H., Choi I. (2018). Binding of Tolperisone Hydrochloride with Human Serum Albumin: Effects on the Conformation, Thermodynamics, and Activity of HSA. Mol. Pharm..

[28] Daimon Y., Izawa H., Kawakami K., Zywicki P., Sakai H., Abe M., Hill J.P., Ariga K. (2014). Media-dependent morphology of supramolecular aggregates of beta-cyclodextrin-grafted chitosan and insulin through multivalent interactions. J. Mater. Chem. B.

[29] Hirose T., Sunazuka T., Omura S. (2010). Recent development of two chitinase inhibitors, Argifin and Argadin, produced by soil microorganisms. Proc. Jpn. Acad. B Phys. Biol. Sci..

[30] Verlee A., Mincke S., Stevens C.V. (2017). Recent developments in antibacterial and antifungal chitosan and its derivatives. Carbohydr. Polym..

