# Supplementary material for: Guanidinylation of Chitooligosaccharides Involving Internal Cyclization of the Guanidino Group on the Reducing End and Effect of Guanidinylation on Protein Binding Ability
Source: Biomolecules. 2019 Jul 5;9(7):259. doi: 10.3390/biom9070259 (PMC6681198; doi:10.3390/biom9070259)
Supplement: Supplementary file 1 [file biomolecules-09-00259-s001.pdf]

biomolecules

Communication

Guanidinylation of Chitooligosaccharides Involving Internal Cyclization of the Guanidino Group on the Reducing End and Effect of Guanidinylation on Protein Binding Ability

Hironori Izawa [1,2,*], Mizuki Kinai [1], Shinsuke Ifuku [1,2], Minoru Morimoto [3] and Hiroyuki Saimoto [1,2,*]

1 Graduate School of Engineering, Tottori University, 4-101 Koyama-Minami, Tottori 680-8550, Japan.
2 Centre for Research on Green Sustainable Chemistry, Tottori University, Tottori 680-8550, Japan.
3 Division of Instrumental Analysis, Research Center for Bioscience and Technology, Tottori University, Tottori 680-8550, Japan.
* Correspondence: h-izawa@tottori-u.ac.jp (H.I.), saimoto@chem.tottori-u.ac.jp (H.S.) ; Tel.: +81-857-31-5813 (H.I.), +81-857-31-5693 (H.S.)

1. ESI-MS Analysis of GCOS

In the ESI-MS spectra, although fragment ion peaks were observed, some detected molecular ion peaks showed good agreement with dehydrated GCOS.

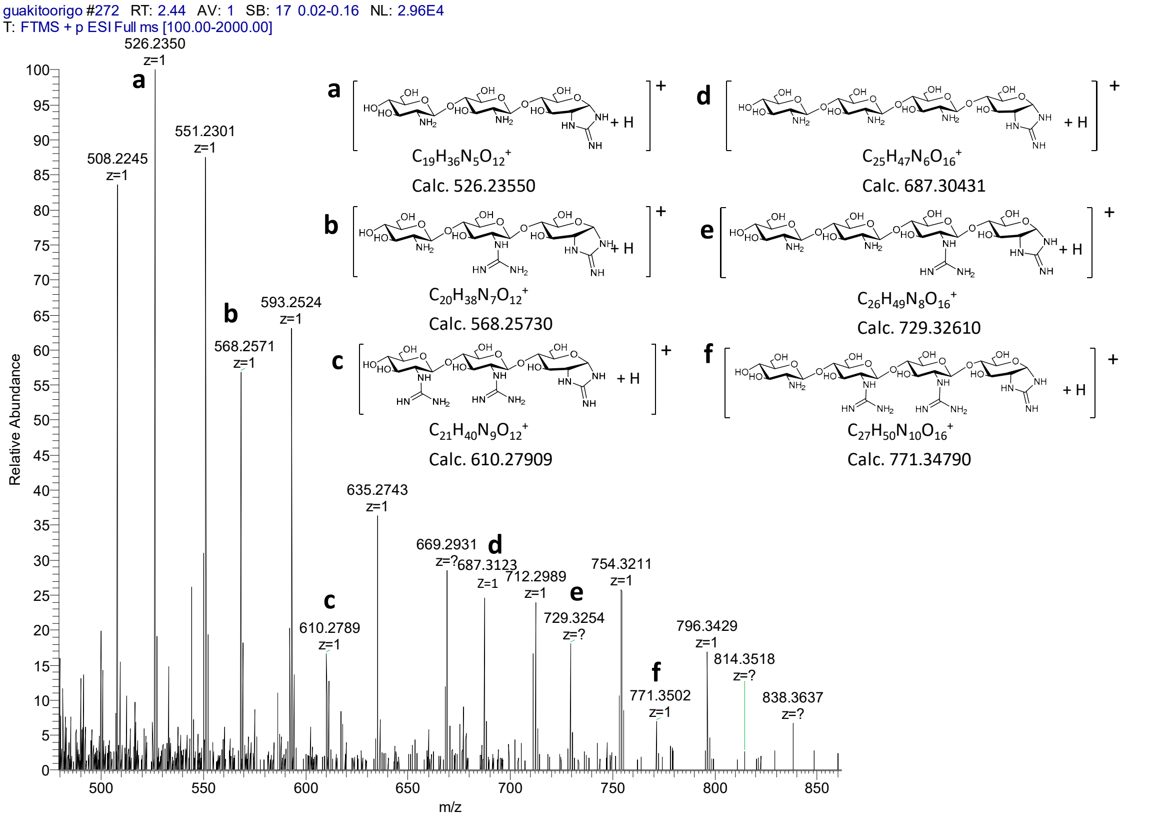

Figure 1. ESI-MS spectrum of GCOS.

2. H-H COSY NMR Analysis of GCOS

Figure S2 shows H-H COSY spectra of GCOS. The correlation of anomeric protons on the reducing end was observed at around 4.6 ppm, indicating that the signal attributed to the proton of the 2-position on the reducing end overlapped with the signals attributed to anomeric protons.

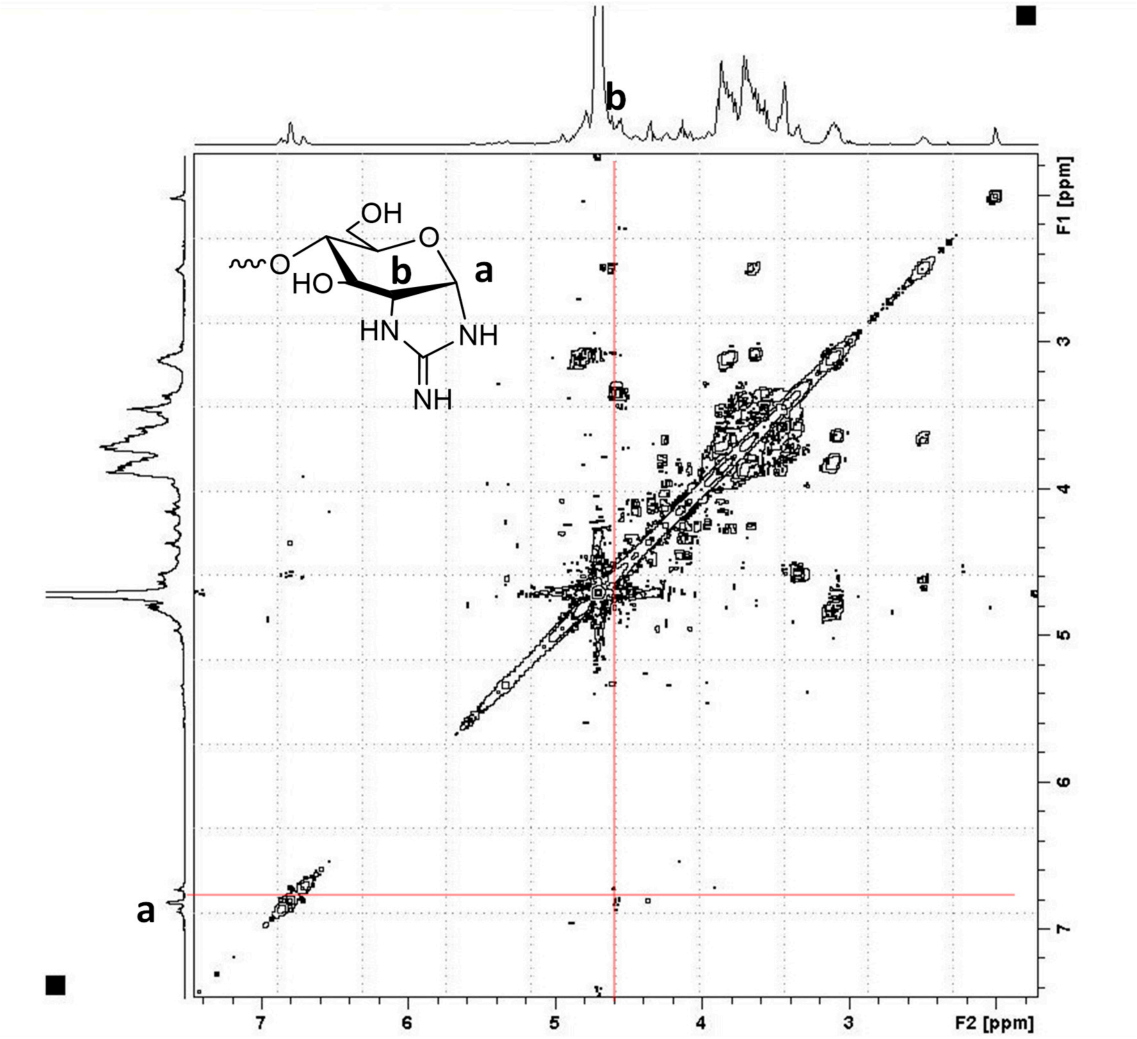

Figure S2. H-H COSY NMR spectra of GCOS in D_2O. The correlation observed at the intersection of the red lines indicates the coupling between H-1 (**a**) and H-2 (**b**) on the reducing end.

3. Stability of the Cyclic Guanidino moiety in Strong Acidic/Basic Condition

Figure S3 shows ^{1}H NMR spectra of GCOS in 1.0 M NaOD and DCl-D_2O solutions after two days. The signal attributed to the anomeric proton on the reducing end at around 6.7 ppm did not disappear. The integration ratios for them also did not change.

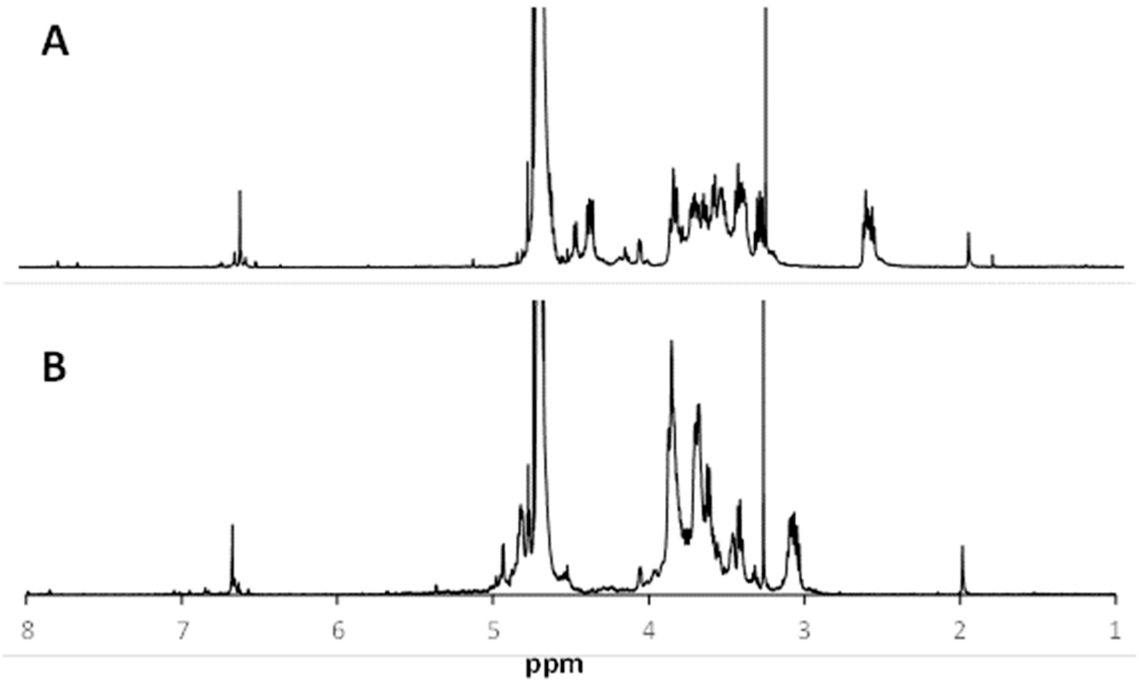

Figure S3. ^{1}H NMR spectra of GCOS in 1.0 M NaOD (**A**) and DCl-D_2O (**B**) solutions after two days.

4. Guanidinylation of GlcN

After guanidinylation, the reaction mixture was lyophilized. The ^{1}H NMR and ESI-MS analyses of the lyophilized mixture were investigated to confirm the production of the cyclic structure. In the ^{1}H NMR spectrum (Figure S4), a signal that was probably due to the anomeric proton in 2-[(aminoiminomethyl)amino]-2-deoxy-D-glucose (CG-GlcN) was observed at 6.64 ppm. In addition, in the ESI-MS spectrum (Figure S5), a molecular ion peak (*m*/*z* = 204.098) corresponding to CG-GlcN (calc. $C_7H_{14}N_3O_4^+$ = 204.10) was detected, suggesting CG-GlcN production. Therefore, in order to purify CG-GlcN, the lyophilized mixture was applied to an acetylation treatment and the acetylated product was purified. Figure S6 shows the ESI-MS spectrum of the acetylated product. Molecular ion peaks corresponding to acetylated CG-GlcN were detected. Figure S7 shows the ^{1}H NMR spectrum of the acetylated product. The signals assigned by H-H COSY NMR analysis (Figure S8) clearly showed the production of acetylated CG-GlcN. These results indicate that GlcN was converted to CG-GlcN by the guanidinylation with AP.

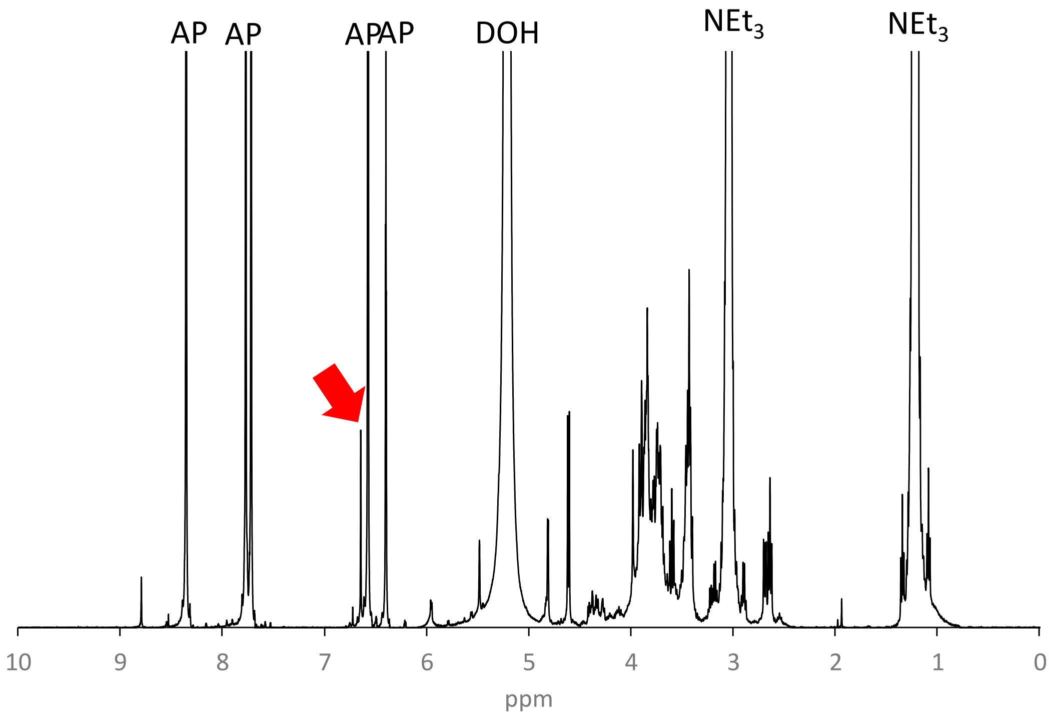

Figure S4. ^{1}H NMR spectrum of the lyophilized mixture in D_2O.

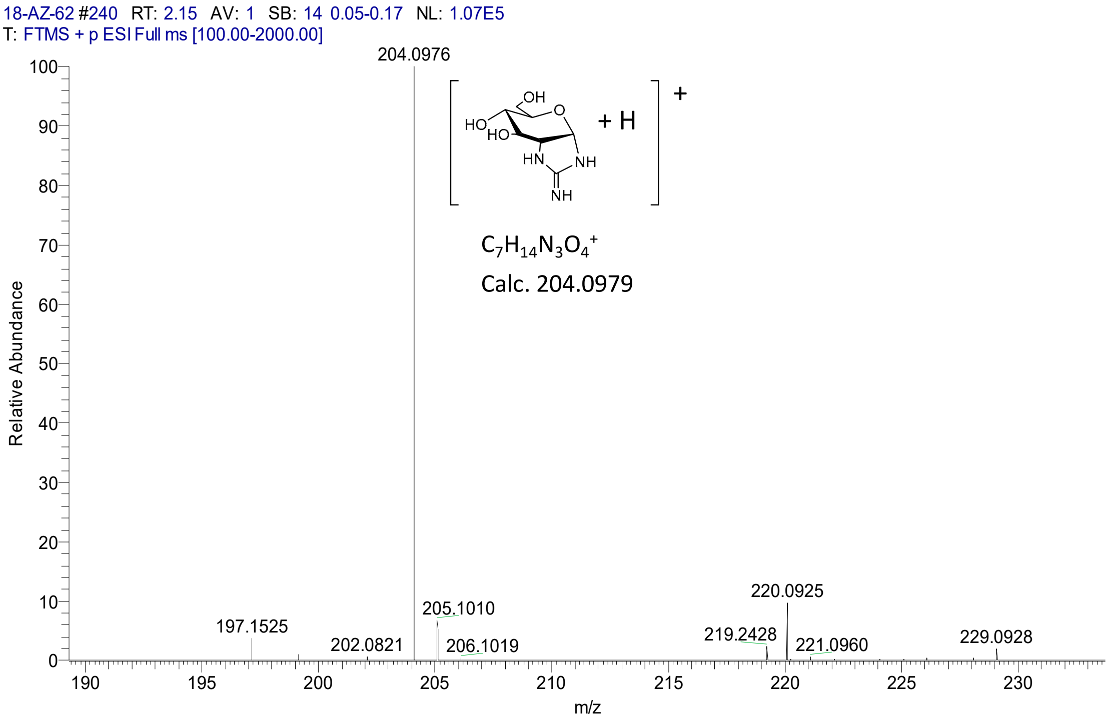

Figure S5. ESI-MS spectrum obtained on the positive mode of the lyophilized mixture.

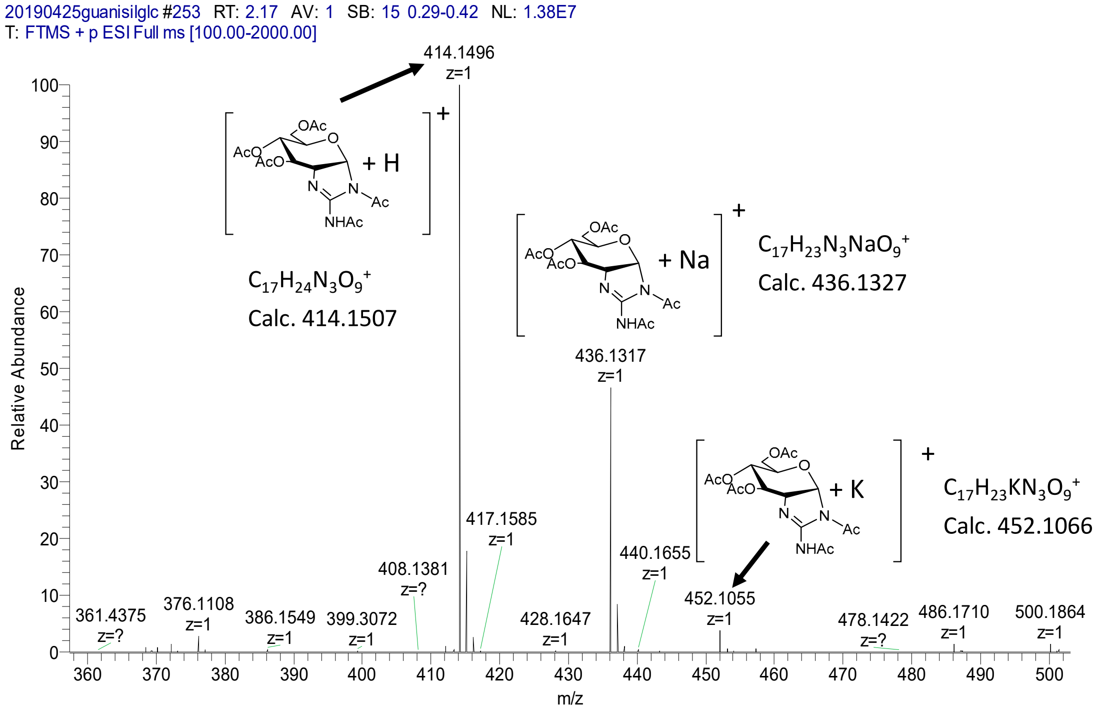

Figure S6. ESI-MS spectrum obtained on the positive mode of the acetylated product.

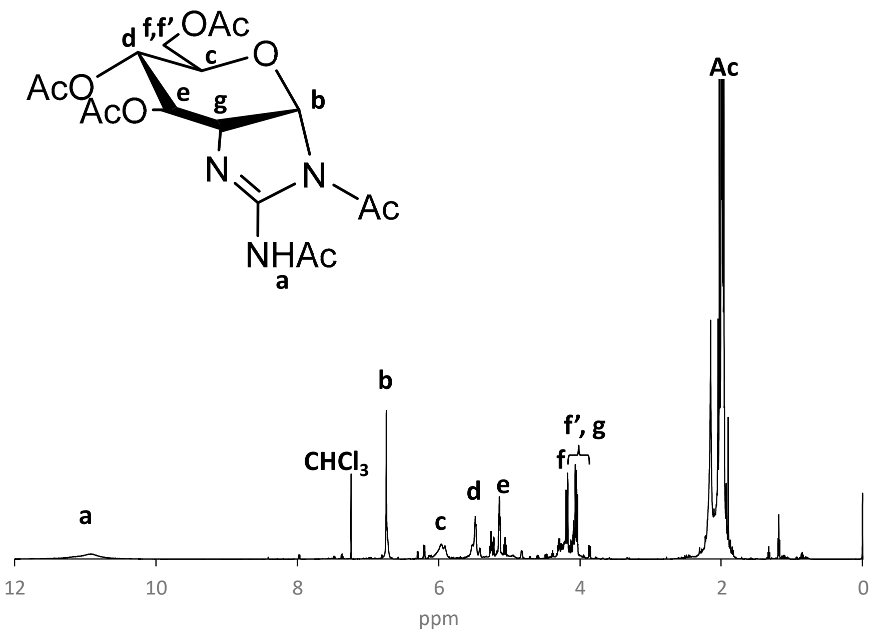

Figure S7. ^{1}H NMR spectrum of the acetylated product in $CDCl_3$.

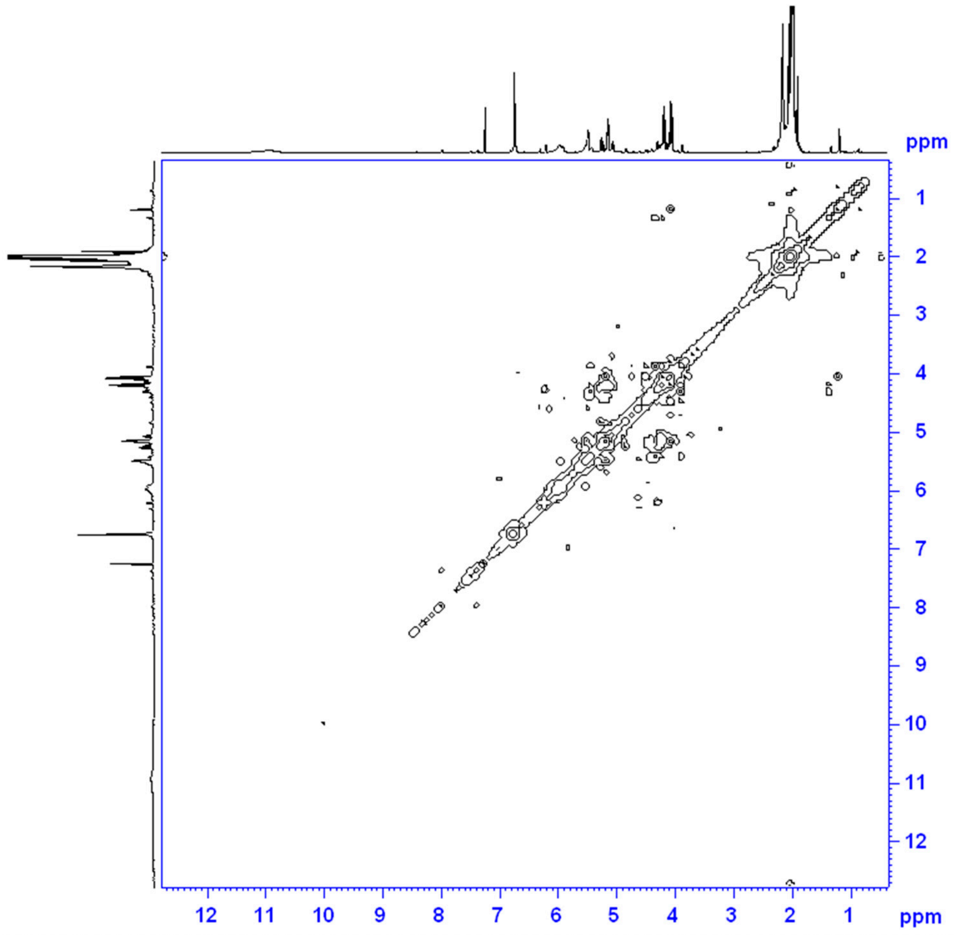

Figure S8. H-H COSY NMR spectra of the acetylated product.

5 The ^{1}H NMR Spectrum of COS Hydrochloride

The signal attributed to the anomeric proton on the reducing end (α-isomer) was shown at 5.4 ppm.

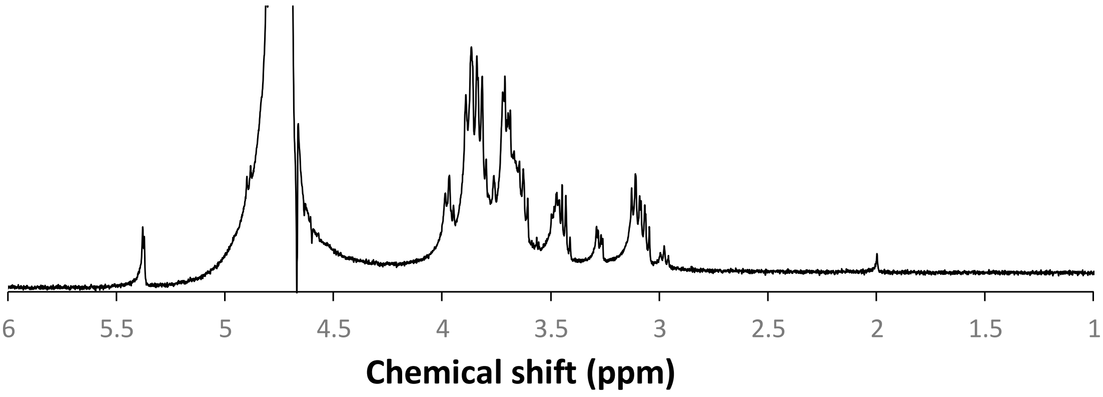

Figure S9. ^{1}H NMR spectrum of COS hydrochloride.

6. Characterization of GCOS Sulfate Salt

Figure S10 shows the infrared (IR) spectrum of GCOS sulfate salt. The absorption peaks were observed at 3163, 1683, 1531m 1376, 1058 cm^{-1}. These peaks perfectly agree with previously reported characteristic absorption peaks observed on the guanidinylated chitosan sulfate salt [1], indicating the production of GCOS sulfate salt. Figure S11 shows the ^{1}H NMR spectrum of GCOS sulfate salt. The signals attributed to anomeric protons on the reducing end was observed at around 6.67 ppm. This is consistent with that of GCOS as shown in Figure 3. The signals attributed to the protons of the two-positions were observed at around 3.09 ppm, which were shifted to lower magnetic field by protonation of amino and guanidine groups.

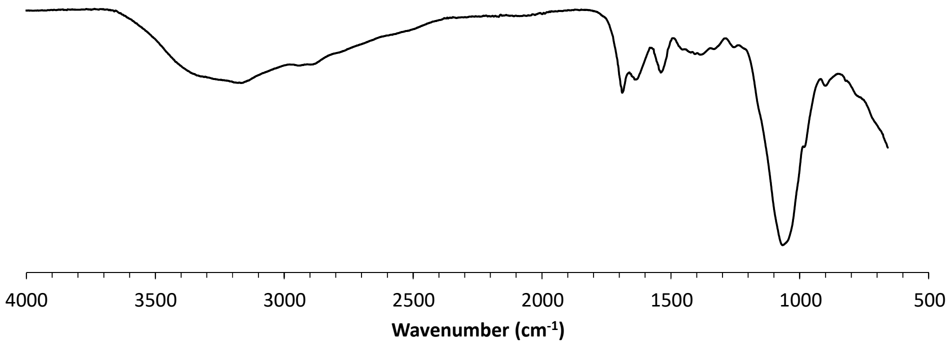

Figure S10. IR spectrum of the GCOS sulfate salt.

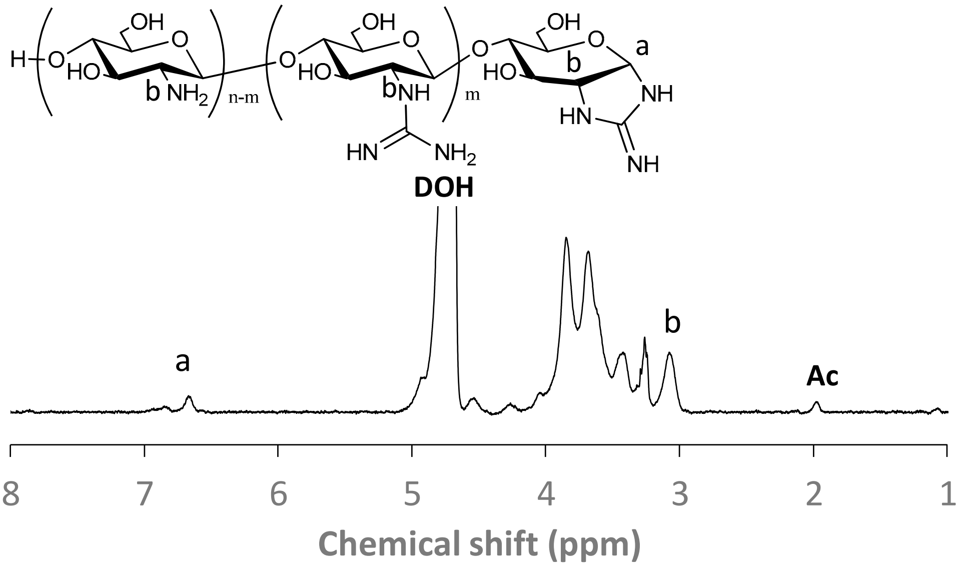

Figure S11. ^{1}H NMR spectrum of the GCOS sulfate salt.

References

1. Hu, Y.; Du, Y.M.; Yang, J.H.; Kennedy, J.F.; Wang, X.H.; Wang, L.S. Synthesis, characterization and antibacterial activity of guanidinylated chitosan. *Carbohyd Polym*. **2007**, *67*, 66–72, doi:10.1016/j.carbpol.2006.04.015.

© 2018 by the authors. Submitted for possible open access publication under the terms and conditions of the Creative Commons Attribution (CC BY) license (http://creativecommons.org/licenses/by/4.0/).
